# Primary adenomyoepithelioma of tonsil

**DOI:** 10.1186/1758-3284-2-7

**Published:** 2010-03-31

**Authors:** Juan Ren, Liping Song, Qiang Dang, Xiaozhi Zhang, Shi-Wen Jiang, Guanjun Zhang, Ning Wang, Zi Liu, Jiansheng Wang, Yi Lisa Hwa, Zongfang Li, Xinhan Zhao, Yuan Liu

**Affiliations:** 1 Cancer center, First Hospital of Xi'an Jiaotong University, Xi'an 710061, Shaan'xi Province, 710061 PR China; 2 Medical school of Xi'an Jiaotong University, Xi'an 710061, Shaan'xi Province, PR China; 3 Department of Basic Biomedical Sciences, Mercer University School of Medicine, GA 31404, USA; 4 Department of Obstetrics and Gynecology, Mayo Clinic, Rochester, MN 55905, USA; 5 Department of Internal Medicine, Mayo Clinic, MN, 55905, USA; 6 Second Hospital of Xi'an Jiaotong University, Xi'an 710061, Shaan'xi Province, 710061 PR China; 7 Department of pathology, Dental Hospital, Fourth Military Medical University, 710038 PR China

## Abstract

We present a case of adenomyoepithlioma (AME) arising from the tonsil. AME is an uncommon tumor that typically arises in breast, but rarely found in salivary glands, lung, and skin. Its biological features have not been thoroughly characterized. Here we describe a primary AME originating from the tonsil. The pathologic changes were characterized by hypercellularity, the dominance of both epithelial and myoepithelial cells. Malignancy was evidenced by the presence of a high mitotic rate and invasive growth. The epithelial cells express high levels of cytokeratin and epithelial membrane antigen (EMA). The myoepithelial cells show positive staining for calponin, p63, vimentin, and S-100. A thorough review of the literature indicates that this is likely the first reported case of AME from the tonsil. Following descriptions of the diagnosis, treatment, and prognosis of this specific case, pathologic and clinical characteristics of AME from other tissues are also compiled and discussed.

## Introduction

Adenomyoepitheliomas (AME) were first described as a rare primary breast tumor by Hamperl in 1970, and fully documented by Kiaer and Eusebi in the mid 1980s. While most adenomyoepithelioma cases were observed in breast, rare cases were found in salivary gland, skin, and lung. Adenomyoepithelioma arising from the tonsil has not been documented in previous literature.

Adenomyoepitheliomas, also known as Epithelial-myoepithelial carcinomas, are considered to be low-grade, malignant neoplasms consisting of a dual population of cells, epithelial cells and myoepithelial cells. These two cells types form bilayered glandular structures, the inner layer of epithelial-type cells and the outer layer of myoepithelial-type cells [1]. The pathological lesion is caused mainly by the deregulated, anaplastic proliferation of epithelial and myoepithelial cells. In this report, we describe an adenomyoepithelioma originating in the tonsil of an elderly woman. We describe the findings from histopathologic studies using multiple diagnostic markers, as well as treatment procedures and differential diagnosis of this particular lesion. Features of adenomyoepitheliomas arising from breast, salivary gland, lung, and skin are also discussed.

## Case report

### Clinical reviews

The patient was a 63-year-old female with an unremarkable medical history for both herself and her family. Her chief complaint was the discomfort in her pharynx and larynx, which had been gradually aggravated for the last 3 months. A fragile, tan neoplasm was found at the lower surface of the left tonsil with the size of 1.0 × 1.0 cm. The neoplasm seemed to bleed easily with touch. No ulceration or mucosal erosion was noted. Hyperplasia of lymphatic tissue was observed in the posterior wall of the pharynx. Biopsy was performed and the histological examination revealed a tumor composed of epithelial and myoepithelial cells. Tonsillectomy was implemented for the left side under local anesthesia. The patient has no history of known malignant disease, and no neoplasm was identified by full-body imaging studies.

### Pathological and immunohistochemical findings

**Figure 1:**
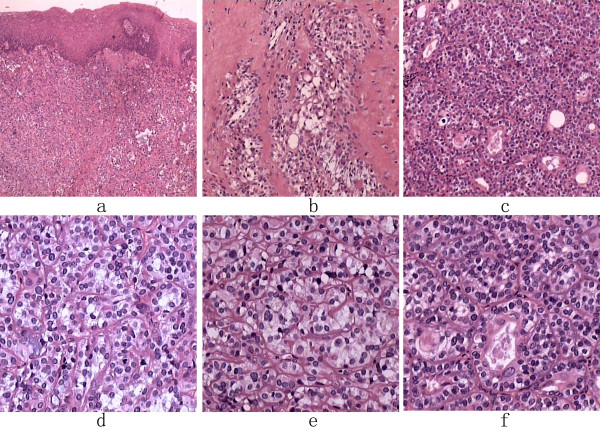
**HE staining** (a), the tumor surface is covered with squamous epithelium cells (40×); (b), tumor cells invaded to the surrounding connective tissues (100×); (c), the structure of the tumor(100×); (d), mitotic phase of the tumor (200×); (e), the structure of the glands (200×); (f), clear cells in the tumor (200×).

The gross appearance of the tumor was a 1.0 × 1.0 cm sized, lobulated neoplasm with an off-white coarser surface. A vertical section of the tumor showed red to gray color. Histologically, the tumor showed bicellular proliferation of both epithelial cells and myoepithelial cells (Fig 1a). Fig 1b shows that the tumor cells invaded into the surrounding tissues, indicating a pattern for invasive growth. Tumor cells are tightly packed, often organized in "glandular" structures (Fig 1C). In addition, Fig 1d shows proliferation of polygonal tumor cells with many mitotic events. Both epithelial cells and myoepithelial cells show pleomorphic cytomplasm and nuclei characteristic of atypia. Atypical cells are enlarged in size, contain irregular nuclei with one or two prominent nucleoli, or show condensed nuclei with dark staining. In the high-power fields, the glandular structure (Fig 1e) and presence of clear cells (Fig 1f) are better observed.

**Figure 2:**
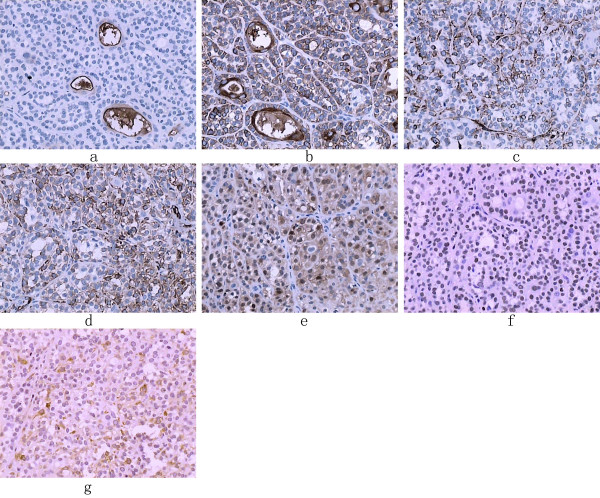
**Immunohistochemical staining, Brown particle was regarded as positive staining signal**. (a), Immunostaining of EMA (200×), EMA antibody was from MaiXin and was 1:80 diluted; (b), Immunostaining of cytokeratin (200×), CK antibody was from MaiXin and was 1:60 diluted; (c), Immunostaining of Vimentin (200×), Vim antibody was from Zhong Shan and was 1:100 diluted; (d), Immunostaining of smooth muscle actin (200×), SMA antibody was from MaiXin and was 1:60 diluted; (e), Immunostaining of S-100 (200×), S-100 antibody was from Zhong Shan and was 1:80 diluted; (f), Immunostaining of P-63 (200×); P-63 antibody was from Zhong Shan and was 1:80 diluted; (g), Immunostaining of Calponin (200×) Cal antibody was from MaiXin and was 1:80 diluted;

Immunohistochemical staining of Fig 2 show the character of biphasic epithelial and myoepithelial cells. EMA and CK are representative markers for epithelial cells. This case is consistent with the presence of such. The glandular epithelial cells positively react to both epithelial membrane antigen (EMA) and cytokeratin (CK) (Fig 2a, b), but were negative for S100 protein, α-smooth muscle actin (SMA), and vimentin (negative staining was not shown). In Fig 2a (EMA staining), the positive staining was only found in gland duct epithelial cells, but not in myoepithelial cells. Cytokeratin is usually expressed mostly in glandular epithelial cells, but occasionally the protein is also expressed in myoepithelial cells. This is seen in our case; CK is most strongly positive in gland duct epithelial cells, but is also positive in myoepithelial cells (Fig 2b showed). In Fig 2c, d, e, f, g, the myoepithelial cells were respectively strongly positive for vimentin, S100 protein, P63 and calponin. Vimentin, SMA and Calponin were seen positive in the cytoplasm of myoepithelial cells in Fig 2c, d and 2g, respectively. Fig 2e shows S-100 highly expressed in both the cytoplasm and nuclei of myoepithelial cells; while P63 was observed positive in the nuclei of myoepithelial cells. Ki-67 labeling was positive and the PCNA positive rate was > 90% (not shown). SMA, calponin, and S-100 are characteristic markers of myoepithelial cells. In our case, myoepithelial cells match this characteristic immunhistochemical feature. The characteristic diagnostic molecules for epithelial cells include EMA and CK, while the representative molecules for myoepithelial cells are SMA, calponin, and S-100. In our case, both myoepithelial cells and epithelial cells express their respective characteristic molecules. In our case, the myoepithelial cells highly express Vimentin, SMA, S100, P63, Calponin, Ki-67 and PCNA. The epithelial cells highly express EMA and cytokeratin, while reacting negatively to S100, SMA and Vimentin. This expression pattern is consistent with the major diagnostic features of adenomyoepithelioma.

### Treatment and prognosis

**Figure 3:**
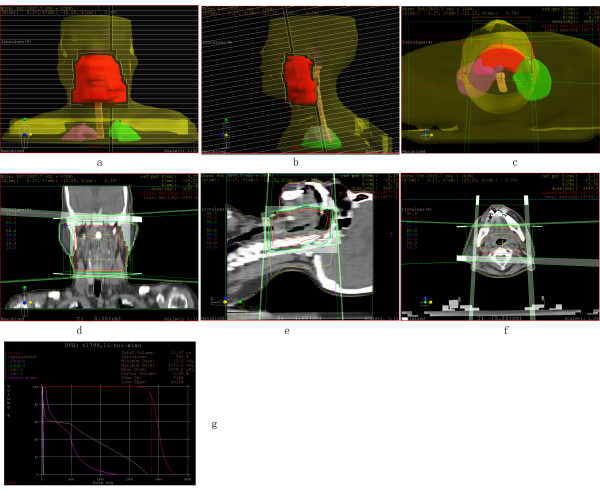
**Plan of Three Dimensional-Conformal Radiation-Therapy**. (a, b, c): The distribution of target region, the target region includes the primary malignancy, pharyngeal lymphoid ring and upper cervical lymph node region; (d, e, f): The distribution of isodose curve, the total tissue dose is 70Gy/35f. The 95% isodose curve covered the planning target volume; (g), The dose-volume histogram showed that the radiation doses received by the surrounding normal tissues, which include brain stem, spinalcord and lens, are all lower than the safe dose threshold.

Given a malignant neoplasm of the tonsil, tonsillectomy was always the first choice of treatment. After the surgery, the pathological diagnosis of adenomyoepithelioma was confirmed based on both light microscope evaluation (routine HE staining) and immunohistochemical analysis using specific antibodies. The patient received a chemotherapy regimen of: Epirubicin 60 mg daily 1 + Cis-platinum 50 mg daily 1~2 + Calcium folinate 200 mg daily 1~5 + Tegafur 800 mg daily 1~5, for 4 cycles. During the chemotherapy, the side effects of digestive system complications (grade I) and marrow suppression (grade II) were observed. After the 4 cycles of systemic chemotherapy, a CT scan of the head and neck showed a slightly thicker oropharyngeal wall than the normal tissue. Multiple enlarged cervical lymph nodes in the carotid triangle were found. Local radiation therapy was subsequently prescribed and the patient received three-dimensional-conformal radiation-therapy. The target region included the tumor bed and regional lymph drainage area. The total tissue dose was 70Gy/35f. The target area, isodose curve, and the dose-volume histogram are shown in Fig 3. During a 1-year follow-up period, there has been no evidence of local recurrence or distant metastasis.

## Discussion

Adenomyoepithelioma (AME) as a diagnostic term has been applied to a broad range of biphasic lesions composed of epithelial and myoepithelial cells. AME is featured as simultaneous proliferations of epithelial and myoepithelial elements. In this case, we found the simultaneous dominancy of epithelial and myoepithelial cells, and both epithelial cells and myoepithelial cells highly express their respective characteristic molecules. The characteristic molecules of epithelial cells include EMA and CK, while the representative markers for myoepithelial cells are SMA, calponin, S-100, and GFAP. The immunohistochemistry staining features distinguish this tumor from a mixed tumor and support the diagnosis as AME.

Depending on the pathologists' preference, other diagnostic terms have been used for AME of different organs. The terms adeno-myoepithelioma, malignant adeno-myoepithelioma, and epithelialmyoepithelial adenoma, and epithelial-myoepithelial carcinoma have the same meaning. But myoepitheliaomas and their malignant counterpart, myoepithelial carcinoma, are different concepts than AME [2].

Myoepitheliomas are tumors composed predominantly or exclusively of myoepithelial cells without ductal (epithelial) development. They are well-described neoplasms of the salivary glands, but such tumors may occur also in the breast and tracheobronchial tree where myoepithelial cells are normally present. Myoepithelial carcinoma is malignant myoepithelioma. There are no definite histological criteria for discriminating benign and malignant myoepitheliomas. As for myoepithelial tumors, nuclear atypia, high mitotic rate and infiltrative growth into adjacent tissues have been proposed as features suggestive of malignant myoepitheliomas. Immunohistochemically, tumor cells are positive for both epithelial markers (EMA and CK) and myogenic markers (alpha-SMA and calponin), variably together with S-100 protein and GFAP. However, it must be noted that the tumor cells are not always positive for these markers, and that negative staining does not necessarily exclude myoepithelial differentiation.

### Common features of adenomyoepithelioma

Ultrastructural features of AME are junctional complexes and apical secretory granules in the periluminal epithelial cells, and basal lamina and myofilaments with focal densities in the myoepithelial cells. The tumors demonstrating a potential for recurrence or metastasis generally had higher mitotic rates, cellular atypia and less well defined margins [3];

Immunohistochemistry staining of AME demonstrates that the myoepithelial cells express S100 protein, Calponin, smooth muscle actin or/and GFAP while not reacting with epithelial markers. The inner epithelial component usually reacts with cytokeratins and EMA, but is non-reactive with S100 and smooth muscle actin. The representative molecules of epithelial cells include EMA and CK, while the characteristic molecules of myoepithelial cells are SMA, calponin, S-100, and GFAP. In our case, both myoepithelial cells and epithelial cells highly express their respective characteristic molecules.

Studies of cell proliferation of salivary AME found that the solid tumors show an overgrowth of myoepithelial-type cells, suggesting that these cells represent the 'proliferative compartment' of tumors. This is in accordance with the observation in breast AME that cell proliferation and aneuploidy are restricted to the myoepithelial-type cell layer, which suggests that the inner layer of epithelial cells represent a more differentiated cell type, probably resulting from differentiation of the myoepithelial type cells.

### Adenomyoepitheliomas arising from different organs

#### Breast adenomyoepithelioma

AME found in breast are much more common than those found in salivary glands, skin, and lung. Some authors have questioned the name "breast adenomyoepithelioma" since these tumors are often histologically identical to and behave in the same fashion as epithelialmyoepithelial carcinomas of salivary gland, lung, or skin. The patient age ranges from 24 to 86 yr, with a mean age of 57 yr [4,5]. Breast AME has been divided into three patterns in terms of histology: spindle-cell, tubular, and lobulated types. Gross examination usually reveals a mass of 0.5 to 10 cm diameter with well-defined or irregular borders. The texture can range from firm, elastic, or rubbery to soft. Papillary or cystic structures may be found in the cut surface. Foci of hemorrhage or necrosis can also be seen, and sometimes calcifications are present. Upon microscopic examination, the tumor can be found to be delineated by a true or pseudo-capsule and is composed of the epithelial and myoepithelial component. Depending on the relative abundance of the two different components, the growth patterns, and the cytological appearances, the tumors can have great variability in histological presentation. The epithelial component may form solid nests or groups, ducts, cystic, rabecular, pseudo-papillary, or papillary structures. Electron microscopy will demonstrate the biphenotypic nature of these cells containing 6-nm actin myofilaments and basal lamina, along with desmosomal structures and perinuclear intermediate filament bundles.

Breast AME has a wide spectrum of cytological features. In the majority of cases, large, tightly cohesive aggregates with a dual population of epithelial and myoepithelial cells were present. The common cytomorphologic features of breast AME include [6]: 1) cellular smears or cohesive sheets containing epithelial and myoepithelial cells; 2) acinar, finger-like, or papillary pattern; 3) abundant bipolar naked nuclei in the background. There are also some uncommon features, such as: 1) spindle cells in groups and singly; 2) atypical epithelial cells. Immunohistochemical studies of breast AME indicated the following features. Cytokeratins such as CAM5.2, CK7, or AE1/3 cocktail highlight the epithelial tubules, with an often more subtle staining of the myoepitheial cells. The myoepithelial component is glycogen-rich, which can be detected by periodic acid schiff's staining. The same type of cells also contains actin that can be recognized by specific antibodies immunohistochemically with S-100 and variably with smooth muscle actin. Muscle specific actin, calponin, and desmin usually stain the myoepithelial cells strongly. Epithelial membrane antigen, p63, cytokeratin 14, CD10, and even glial fibrillary acidic protein3 have also been shown to be present in myoepithelial cells.

Although most of breast AMEs are considered benign, they can recur locally, or progress subsequently to a malignant state and give rise to metastases. Malignant transformation may involve epithelial cells, myoepithelial cells, or both cellular elements [7]. According to the most recent World Health Organization (WHO) classification, malignant AME of the breast includes: 1) myoepithelial carcinoma arising in an AME; 2) epithelial carcinoma arising in an AME; 3) malignant epithelial and myoepithelial components; 4) sarcoma arising in AME; 5) carcinosarcoma arising in AME [8].

#### Adenomyoepithelioma of salivary glands

AME of the salivary glands resembles AME of the breast in their histological presentation. Both form double layered structures, the inner layer of epithelial-type cells and the outer layer of myoepithelial-type cells. Despite these similarities, AMEs of salivary glands belong to a different entity by the following consideration. First, AME is a very distinctive and rare tumor type of salivary glands. AMEs of salivary glands are also invariably malignant, a stark contrast to AME of the breast. However, in both organs, the possible end-spectrum of the disease is myoepithelial carcinoma. Second, Tavassoli reported differences in glial fibrillary acidic protein (GFAP) reactivity in the myoepithelial component of the two tumors. The myoepithelial cells in salivary gland AME sometimes stain for GFAP, whereas the myoepithelial cells in breast AMEs do not.

#### Lung adenomyoepithelioma

Pulmonary AMEs with epithelial and myoepithelial differentiation are rare, thought to be of bronchial minor salivary gland origin and classified similarly to salivary gland neoplasms [9-11]. Such tumors presented with single or multiple pulmonary nodules. Histologically, these tumors showed glandular structure and spindle cell differentiation [12]. Some glands were filled with colloid like secretion and had an inner, cuboidal epithelial cell layer that stain positive for pankeratin, epithelial membrane antigen, cytokeratins (CAM 5.2, CK7), SP-A, and thyroid transcription factor-1(TTF-1), but negative for high molecular weight keratin and myoepithelial markers. There may also be glands lined by a single layer of plump cells that were positive for surfactant protein-A and other epithelial cell markers. The outer layer of myoepithelial cells merge with foci of spindled myoepithelial cells stained positive for high molecular weight keratin, S100, smooth muscle actin, calponin, caldesmon, and p63, but negative for desmin. In addition, they were also strongly positive for CK7 and weakly positive for CAM 5.2 and TTF-1. Neuroendocrine markers (neuron-specific enolase, synaptophysin, chromogranin) and thyroglobulin gave negative results. Alveolar entrapment was excluded by histologic appearance and by complete absence of elastic fibers within the tumor. Electron microscopy confirmed pneumocytic features in these cells and the myoepithelial nature of the spindled cells [13].

#### Skin adenomyoepithelioma

Tumors of skin AME were also composed of epithelial cells and myoepithelial cells. The myoepithelial cells contained myofilaments with focal densities and hemidesmosomes [14,15]. They were limited by a well-formed basal lamina. Immunohistochemically, the epithelial cells exhibited strong expression of cytokeratin (CAM5.2) and weak expression of carcinoembryonic antigen. The myoepithelial cells showed diffuse positive staining for smooth muscle actin and focal positive for S100 protein.

We herein reported a very rare case of malignant AME from tonsil. Based on the pathological characteristics, we offer a diagnostic reference for classifying AMEs from tonsil. This report also provides the treatment options including chemotherapy and local radiation therapy plans. Long-term clinical follow-up appears to be necessary for better understanding of this disease.

## Competing interests

The authors declare that they have no competing interests.

## Authors' contributions

JR, QD, SJ, LS, and NW performed the histological, pathological and immunohistochemical study. GZ and YL carried out the immunostaining. JR, QD, SJ, LS, XZ, NW and YH did the literature review and participate the writing. JR, LS, XZ, NW, ZL, JW, ZL and XZ participated in the patient treatment. All authors read and approved the final manuscript.
