# Primary adenomyoepithelioma of tonsil

**DOI:** 10.1186/1758-3284-2-17

**Published:** 2010-07-09

**Authors:** Juan Ren, Liping Song, Qiang Dang, Xiaozhi Zhang, Shi-Wen Jiang, Guanjun Zhang, Ning Wang, Zi Liu, Jiansheng Wang, Yi Lisa Hwa, Zongfang Li, Xinhan Zhao, Yuan Liu

**Affiliations:** 1 Cancer center, First Hospital of Xi'an Jiaotong University, Xi'an 710061, Shaan'xi Province, 710061 China; 2 Medical school of Xi'an Jiaotong University, Xi'an 710061, Shaan'xi Province, China; 3 Department of Basic Biomedical Sciences, Mercer University School of Medicine, GA 31404, USA; 4 Department of Obstetrics and Gynecology, Mayo Clinic, Rochester, MN 55905, USA; 5 Department of Internal Medicine, Mayo Clinic, MN, 55905, USA; 6 Second Hospital of Xi'an Jiaotong University, Xi'an 710061, Shaan'xi Province, 710061 China; 7 Department of pathology, Dental Hospital, Fourth Military Medical University, 710038 China

## Correction

In our publication [1] we used several points and sentences from other publications and failed to cite a few important sources. Ning Wang, who prepared the first draft of the manuscript, and Dr. Juan Ren, the corresponding author, would like to apologize to the authors of those publications as well as the other authors of this article. They would like to make this correction by citing the appropriate references as listed below.

In the **"Discussion" **section, the third sentence of the third paragraph should have included the citation as follows:

There are no definite histological criteria for discriminating benign and malignant myoepitheliomas. As for myoepithelial tumors, nuclear atypia, high mitotic rate and infiltrative growth into adjacent tissues have been proposed as features suggestive of malignant myoepitheliomas. Immunohistochemically, tumor cells are positive for both epithelial markers (EMA and CK) and myogenic markers (alpha-SMA and calponin), variably together with S-100 protein and GFAP. However, it must be noted that the tumor cells are not always positive for these markers, and that negative staining does not necessarily exclude myoepithelial differentiation [2].

In the **"Common features of adenomyoepithelioma" **section, the second sentence of the third paragraph should have include this citation:

This is in accordance with the observation in breast AME that cell proliferation and aneuploidy are restricted to the myoepithelial-type cell layer, which suggests that the inner layer of epithelial cells represent a more differentiated cell type, probably resulting from differentiation of the myoepithelial type cells [3].

In the "**Breast adenomyoepithelioma" **section, the following citations should have been should be referenced accordingly.

The eight sentence of the first paragraph should have included the following reference:

Depending on the relative abundance of the two different components, the growth patterns, and the cytological appearances, the tumors can have great variability in histological presentation [4].

The tenth sentence in the first paragraph should have been referenced:

Electron microscopy will demonstrate the biphenotypic nature of these cells containing 6-nm actin myofilaments and basal lamina, along with desmosomal structures and perinuclear intermediate filament bundles [5].

The fifth sentence in the second paragraph should also cite this reference:

Cytokeratins such as CAM5.2, CK7, or AE1/3 cocktail highlight the epithelial tubules, with an often more subtle staining of the myoepitheial cells [5].

The eighth sentence in the second paragraph should also be referenced:

Muscle specific actin, calponin, and desmin usually stain the myoepithelial cells strongly. Epithelial membrane antigen, p63, cytokeratin 14, CD10, and even glial fibrillary acidic protein3 have also been shown to be present in myoepithelial cells [5].

In the section **"Lung adenomyoepithelioma" **the third sentence should have included the citation as follows:

Some glands were filled with colloid like secretion and had an inner, cuboidal epithelial cell layer that stain positive for pankeratin, epithelial membrane antigen, cytokeratins (CAM 5.2, CK7), SP-A, and thyroid transcription factor-1(TTF-1), but negative for high molecular weight keratin and myoepithelial markers [6].
